# Temperature-dependent Crystallization of MoS_2_ Nanoflakes on Graphene Nanosheets for Electrocatalysis

**DOI:** 10.1186/s11671-017-2248-9

**Published:** 2017-08-04

**Authors:** Xiaoru Guo, Yang Hou, Ren Ren, Junhong Chen

**Affiliations:** Department of Mechanical Engineering, University of Wisconsin-Milwaukee, 3200 North Cramer Street, Milwaukee, WI 53211 USA

**Keywords:** Crystallization, MoS_2_/graphene hybrids, Electrocatalysis, Hydrogen evolution, Temperature-dependence

## Abstract

This work primarily studies the crystallization condition of molybdenum disulfide (MoS_2_) in MoS_2_/graphene hybrids by a temperature-varying hydrothermal method from 150 to 240 °C. Flower-like MoS_2_ nanoflakes were successfully grown on graphene nanosheets and characterized to understand the temperature-dependent crystallization process and the electrochemical performance. The highest electrocatalytic efficiency for both the dye-sensitized solar cell and the hydrogen evolution reaction was obtained by preparing the hybrid at 180 °C, which benefits from balanced high reactivity and high conductivity. This research leads to a better understanding of temperature dependence of MoS_2_ crystallization and offers guidelines for better catalytic material design.

**Electronic supplementary material:**

The online version of this article (doi:10.1186/s11671-017-2248-9) contains supplementary material, which is available to authorized users.

**Graphical abstract:**
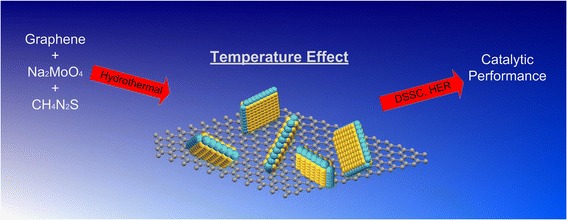
Temperature-dependent Crystallization of MoS_2_ Nanoflakes on Graphene Nanosheets for Electrocatalysis

## Background

Two-dimensional (2D) hybrid materials have been studied for use in photovoltaics, water splitting, sensors, batteries, and many other applications, often in the form of heterojunctions or three-dimensional (3D) frameworks [1–6]. Benefiting from their unique 2D structures and tunable band-gaps, 2D hybrid materials can offer both a high specific surface area and a suitable work function [1, 7–10]. For most electrochemical applications, such as in dye-sensitized solar cells (DSSCs) and hydrogen evolution reaction (HERs), the high electronic conductivity and the strong redox reactivity of transition metal dichalcogenides (TMDs)/graphene hybrids are extremely attractive. In these hybrids, graphene nanosheets possess high electronic conductivity, mechanical strength [11, 12], and serve as growth centers for TMD nanosheets. Earlier studies have shown that the hybrid structures offer enhanced catalytic activity with more active sites [13].

Compared with traditional platinum (Pt)-based catalyst materials, 2D hybrid materials offer a comparable performance and a much lower production cost, thus demonstrating their great potential for replacing Pt for commercial use. Until now, the MoS_2_/graphene hybrid has been studied as one of the most promising options because of its excellent electrocatalytic activity and unique 2D structure [3, 14, 15]. It is well known that poor intrinsic conductivity limits the overall electrocatalytic performance of pure MoS_2_ [16, 17] and that the reactivity of pure graphene is relatively weak [18–20]. The MoS_2_/graphene hybrid combines the benefits of reactivity and conductivity of the two constituent materials, thereby leading to significantly enhanced electrocatalytic performance [21, 22]. In a hydrothermal process, graphene nanosheets also serve as the crystallization core for the MoS_2_ formation to improve the production rate [23–27]. Because both the composition and the structure of catalysts affect the material reactivity, it is important to create more active sites and to maintain high conductivity when designing a hybrid. By choosing appropriate methods to tune the binding between the two component structures, the resulting catalytic performance can be further optimized.

To create the hybrid, many approaches have been explored and their advantages have been compared. Dai’s group prepared the heterojunction of MoS_2_ and graphene through a hydrothermal reaction in organic solvents and explored the kinetics of catalytic reactions [12]. Zhang et al. studied controlled chemical vapor deposition growth of MoS_2_ onto graphene and highlighted the effect of coverage factor [28]. In recent years, hydrothermal methods have been widely studied as a low-cost and high-throughput route for fabricating MoS_2_/graphene hybrids [12, 26, 29–32]. Previous research has reported that the crystallization of pure MoS_2_ could change significantly with different reaction temperatures, with amorphous MoS_2_ nanospheres at low temperatures (120–150 °C), flower-like MoS_2_ balls with a high catalytic performance at mid-range temperatures (160–240 °C), and large MoS_2_ nanoparticles at high temperatures (230–260 °C) [33, 34]. However, when the seed of crystallization changes to graphene, the crystallization condition of MoS_2_ is not well understood, and thus further understanding of the crystallization condition is essential to optimize the material catalytic activity. In this work, we report a facile hydrothermal method to prepare MoS_2_ nanoflakes grown onto graphene nanosheets at different mid-range temperatures. MoS_2_ crystallization on graphene nanosheets can be clearly identified by various crystal characterization methods, and the effects of the crystallization on the resulting catalytic performance are studied by DSSC performance and HER reactivity.

## Methods

### Material Preparation and Characterization

Various MoS_2_/graphene hybrids were prepared by the hydrothermal method (details in the Supporting Information). First, microwave-exfoliated graphene oxide nanosheets (MEGO) were prepared from graphite oxide under an argon environment with exposure to 900 W microwaves for 90 s [35]; this process also reduced the graphene oxide [25]. Then, 2.8 mg MEGO was dispersed in 20 mL DI water by ultrasonication, followed by dissolving 42 mg sodium molybdate dihydrate and 84 mg thiourea sequentially. Excessive thiourea was added to the solution to further reduce MEGO [3]. The suspension was then transferred to 50 mL autoclaves for hydrothermal reactions at temperatures of 150 °C (MG-150), 180 °C (MG-180), 210 °C (MG-210), and 240 °C (MG-240) for 24 h. Finally, the obtained solids were separated, washed, and dried under vacuum at 70 °C overnight.

The structure of prepared materials was studied with a Hitachi (S-4800) field-emission scanning electron microscope (FE-SEM). The energy-dispersive X-ray spectroscopy (EDS) mapping data were obtained using a Bruker detector on a Hitachi S-4800. A Hitachi (H 9000 NAR) system was used to take transmission electron microscope/high-resolution transmission electron microscope (TEM/HRTEM) and to study the hybrid junction of the MoS_2_/graphene hybrid prepared at 180 °C. X-ray diffraction (XRD) was done using a Bruker D8 Discover X-ray diffractometer. Raman spectroscopy was taken with a Renishaw Raman spectrometer (Inc 1000B) with an HeNe laser (633 nm). X-ray photoelectron spectroscopy (XPS) was studied through VG ESCA 2000 with Mg, Kα as X-ray source, and peaks are calibrated with C1s peaks at 284.6 eV.

### DSSC Fabrication and Tests

First, FTO glasses were sequentially cleaned with acetone, isopropyl alcohol, and DI water. Following earlier publications [36], a TiO_2_ nanoparticle structure was formed, by doctor-blading a commercial TiO_2_ paste and gradually heating to 500 °C over 30 min. After the treatments, the substrates were transferred to 0.5 mM N719 ethanol solution and were soaked for 24 h. The counter electrodes were also fabricated by doctor-blading. The slurry contains 20 mg sample and 5 μL Triton ×100 in 500 μL DI water. After coating, the electrodes were annealed at 500 °C for 30 min in an argon environment. Pt-based counter electrodes were fabricated by blading 0.01 M H_2_PtCl_6_ ethanol solution with the same steps. To assemble the cell, the prepared counter electrodes and photoanodes were sealed with a commercial thermoplastic sealing film, and then a commercial electrolyte was injected into the cell.

The J-V characterization was conducted under a simulated one sun illumination (AM 1.5G, 100 mW/cm^2^, Newport, 94021A) with a Keithley 2420 source meter. The system was calibrated with a Si-reference cell (Oriel, P/N 91150V). The electrochemical impedance spectroscopy (EIS) of DSSCs was tested at a frequency from 0.1 to 10,000 Hz, under one sun illumination. The potential was set at 0.7 V, which is about the average open circuit voltage. The data was recorded by a CHI 760D electrochemical workstation.

### Electrochemical Measurements

A saturated Ag/AgCl reference electrode was used in all measurements and was converted to the reversible hydrogen electrode (RHE) scale via the Nernst equation. All measurements were carried out in 0.5 M H_2_SO_4_ aqueous solution using a CHI 760D electrochemical workstation. Tests were performed in a standard three-electrode glass cell, with the Pt wire as the counter electrode and glassy carbon electrode (GCE). To fabricate GCEs, 5 mg of material was mixed with 50 μL Nafion ethanol solution (5%) and 450 μL DI water. The mixture was well dispersed and a 5 μL suspension was dropped onto a glassy carbon electrode with a diameter of 3 mm and then fully dried.

The linear sweep voltammetry (LSV) was tested from 0.2 to −0.8 V (vs. Ag/AgCl) at 5 mV/s; later the Tafel plot was calculated from LSV. Cyclic voltammetry (CV) was scanned between −1 V and 1 V (vs. Ag/AgCl) at 0.05 V/s. The electrochemical impedance spectroscopy was measured at a frequency ranging from 0.1 to 10,000 Hz at a constant potential 0.5 mV (vs. Ag/AgCl). The stability was evaluated for 20,000 s at a constant potential −0.5 V (vs. Ag/AgCl).

## Results and Discussion

**Fig. 1:**
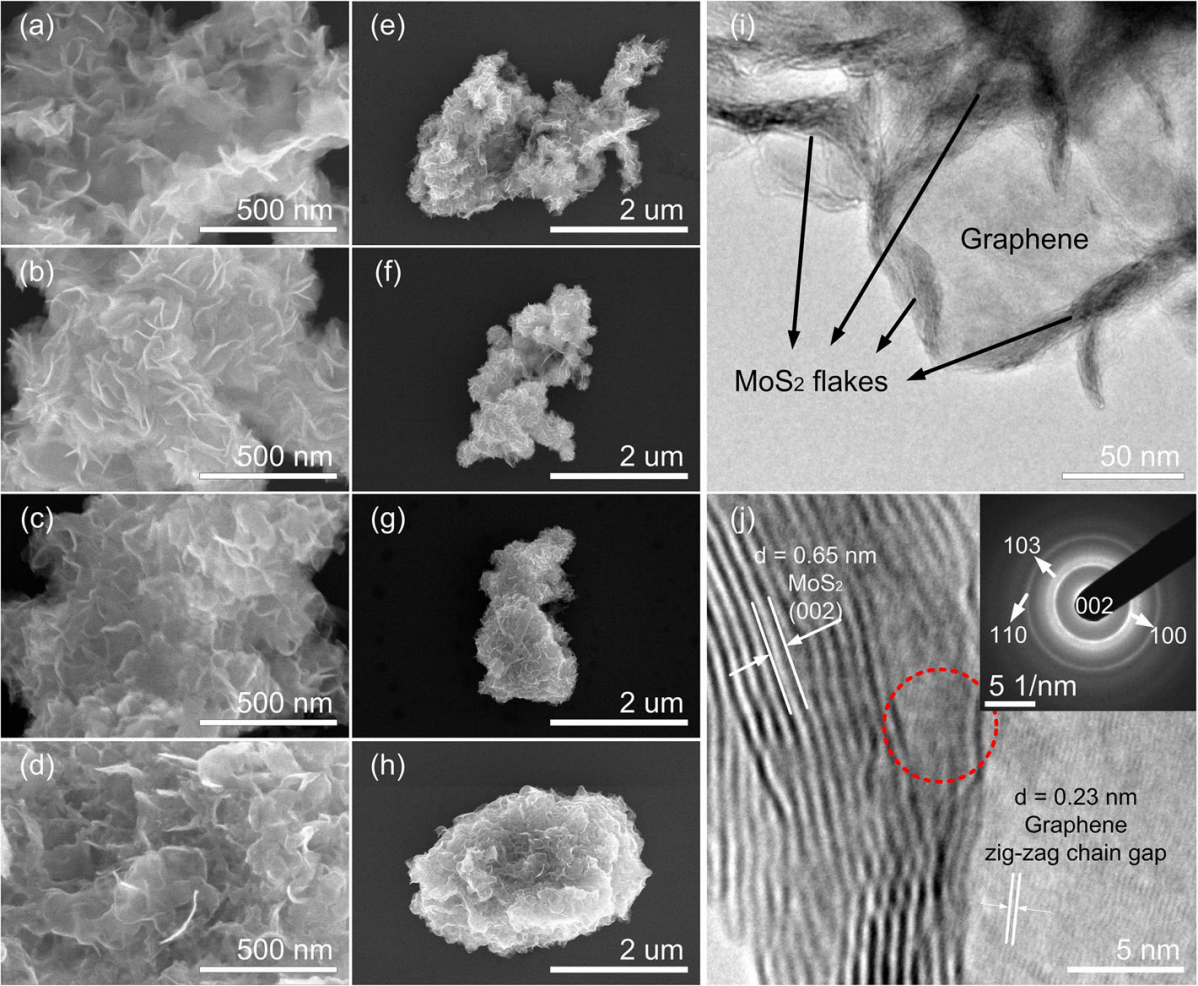
Morphology of MoS_2/_graphene hybrids. SEM images of MoS_2/_graphene hybrids at 150 °C (**a**, **e**), 180 °C (**b**, **f**), 210 °C (**c**, **g**), 240 °C (**d**, **h**), and TEM and HRTEM images of the MoS_2_/graphene hybrid at 180 °C (**i**, **j**). The *inset* of (**j**) is the corresponding SAED pattern marked by the dashed circle. Lattice information is marked in (**j**)

Figure 1a–h shows FE-SEM images of the MoS_2_ structure grown on the graphene surface. The perpendicularly oriented, flower-like MoS_2_ nanoflakes were observed at all temperatures and the uniform coverage was proven by EDS (Supporting Information, Additional file 1: Figure S1). As shown in Fig. 1 a–d, the size of MoS_2_ nanoflakes grew bigger with the increasing synthesis temperature. Observed under a low magnification as shown in Fig. 1e–h, the coverage of MoS_2_ nanoflakes is significantly larger, as the MG-240 hybrid started losing the layer-by-layer feature and began forming the nanoparticles, while the MG-210 hybrid loosely maintained the layered structure. Previous studies have shown that the edges of nanosheets are active sites for catalytic reactions, suggesting that edges, defects, and kinks are responsible for high catalytic performance. Therefore, highly branched morphology is preferred for most catalytic applications [2, 37].

To gain further insights into MoS_2_/graphene hybrids, TEM and HRTEM images were obtained and analyzed. Using an MG-180 hybrid sample to study its branch structure, a laminar structure of MoS_2_ (crossing black stripes) loaded on the surface of graphene (a flat gray area) was observed, as shown in Fig. 1i. Zooming to the center of Fig. 1i, two different types of crystals are clearly observed in the HRTEM image by their significantly different lattice spacings (Fig. 1j). The lattice spacing of 0.65 nm matches well with that of MoS_2_ in 2H–crystal (002) face, and the 0.23 nm lattice spacing is close to that of the zig-zag chain gap in a single-layer graphene nanosheet [38]. The few-layer MoS_2_ nanosheets crossed over to each other in the small area, representing the formation of small nanoflakes and the creation of edges and defects. The seamless stitching of the graphene nanosheet to MoS_2_ nanosheets, marked by the dashed circle in Fig. 1j, also was studied by selected area electron diffraction (SAED). Several diffraction rings can be well-indexed to the planes of 2H–MoS2, with graphene diffraction barely shown due to the small fraction of graphene and strong background of amorphous carbon. The intimate contact of the two types of crystals suggests an efficient electron transfer within the hybrid. A comparison of HRTEM and SAED images of all four hybrids is also shown in Additional file 1: Figure S2. The crystallization significantly improves with the increasing temperature.

**Fig. 2:**
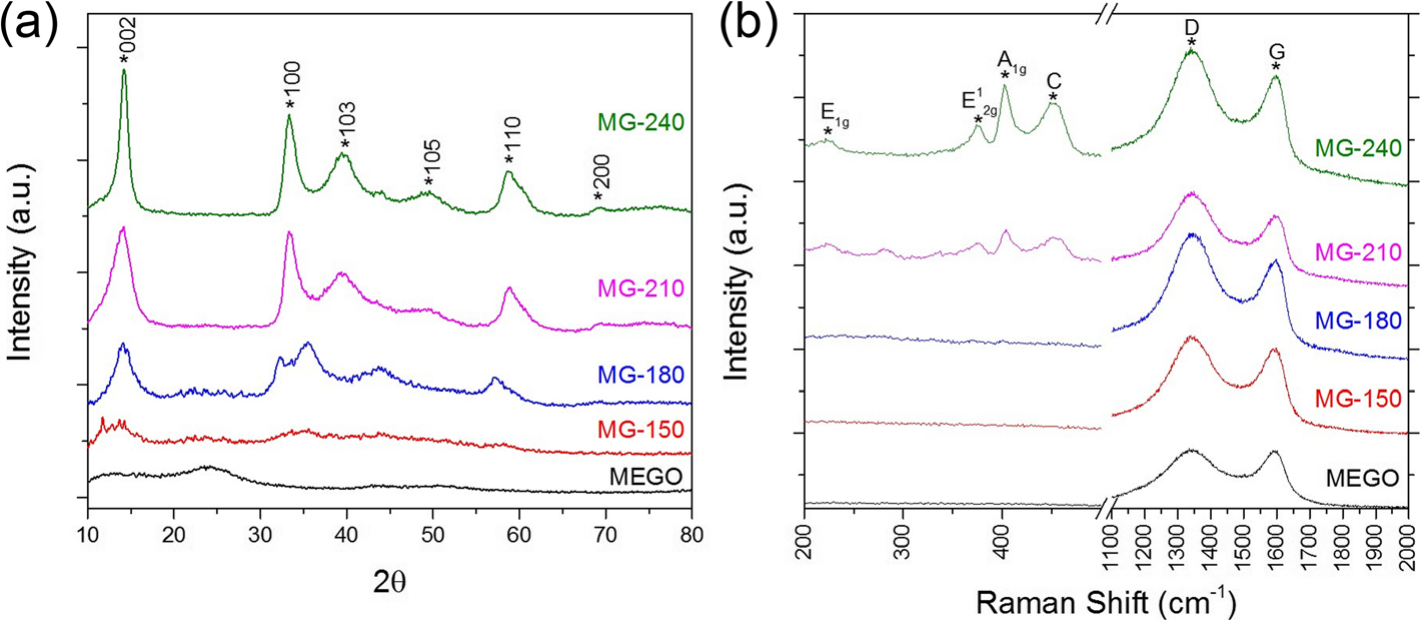
Crystallization comparison of MoS_2_/graphene hybrids. **a** XRD spectra of MoS_2_/graphene hybrids prepared at 150, 180, 210, and 240 °C compared with MEGO, (**b**) Raman spectra of MoS_2_/graphene hybrids and MEGO. 2H peaks of MoS_2_ are labeled in the patterns

To gain deeper understanding of the crystallization change at different reaction temperatures, the XRD and Raman spectra of the MoS_2_/graphene hybrids (Fig. 2) were studied. Overall, the as-prepared hybrids showed a 2H–MoS_2_ phase. The flat XRD pattern from 10^o^ to 35^o^ of MEGO was caused by the stacking of nanosheets while in storage. For the MG-150, MoS_2_ peaks were not clearly visible because of the limited amount of crystal formation on the graphene nanosheets. When the temperature was increased, the XRD peaks sharpened and a small angle shift was observed between 30^o^ and 55^o^. The peaks of the MG-180 hybrid stand out due to the weak peaks for (103) and (105) of the 2H phase, the broadened and shifted (100) peak, and, importantly, an additional (006 + 104) peak. The rearrangement in the crystals indicates possible 1 T phase existence [39]. The weak signals from the MG-150 suggested poor crystallization quality and the presence of rich defects. Similar trends also can be observed by Raman spectra (Fig. 2b) with helium-neon laser excited at 633 nm. Both MG-150 and MG-180 exhibited extremely weak MoS_2_ Raman signatures, which suggest poor crystallization quality. The intensity of A_1g_, E_2g_^1^, and E_1g_ peaks increased with the increasing temperature. Also, the out-of-plane Mo-S phonon mode (A_1g_) is preferentially excited for the edge-terminated perpendicular orientation of MoS_2_ nanosheets, and the high intensity of A_1g_ shown in the MG-210 and MG-240 hybrids indicates the perpendicularly oriented structure formed on graphene nanosheets [2]. The C peaks come from the second order longitudinal acoustic mode at the M point (2LA(M)) of the MoS_2_ Brillouin zone, which indicates improved crystallization quality at a high temperature [40]. Another interesting observation is the increased intensity of the D to G band (I_D_/I_G_) of graphene with increasing temperatures, as shown in Fig. 2b. This indicates a stronger van der Waals interaction between MoS_2_ nanosheets and graphene nanosheets, which enhanced the breathing mode of the hexagonal ring of graphene.

**Fig. 3:**
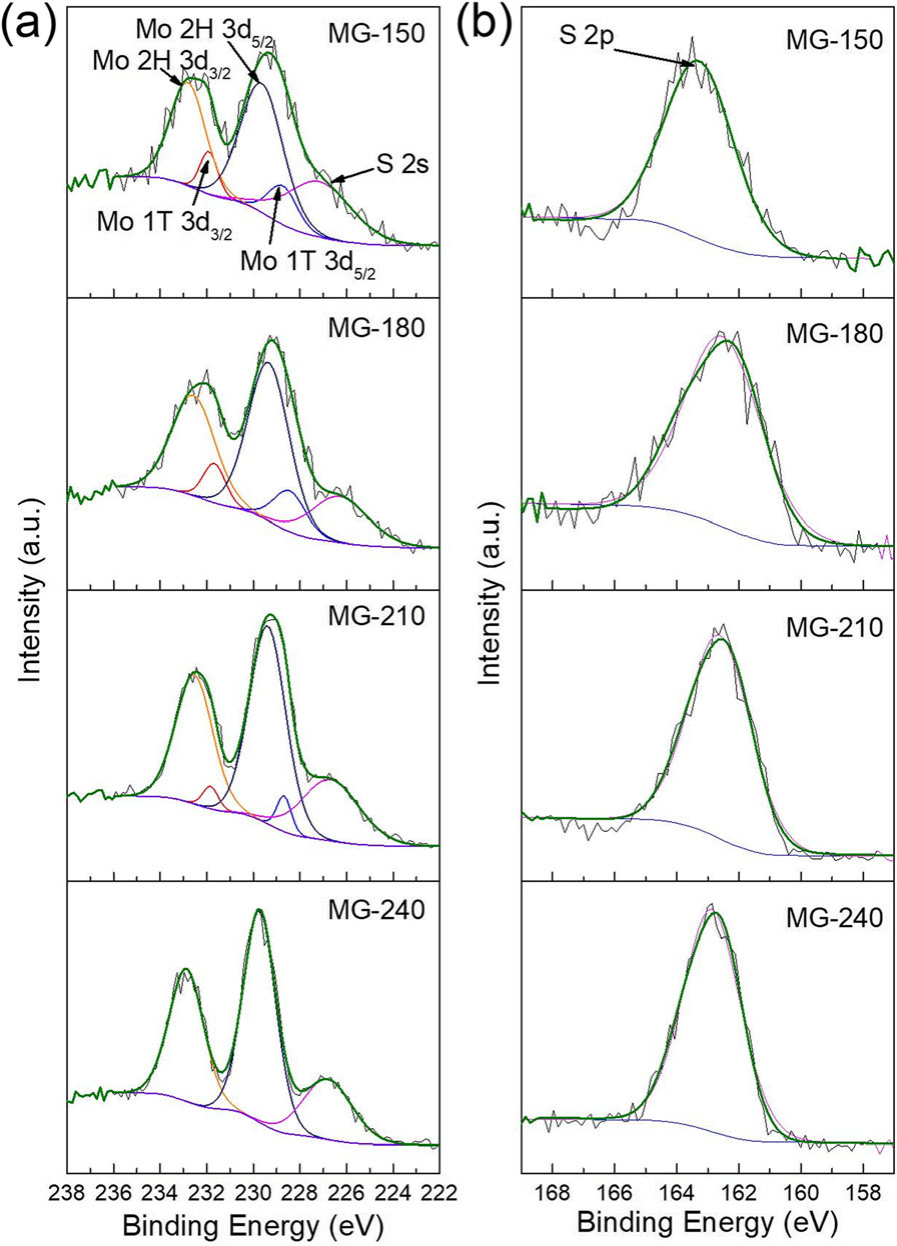
Binding analysis of MoS_2_/graphene hybrids. XPS spectra of MoS_2_/graphene hybrids prepared at 150, 180, 210, and 240 °C, with (**a**) focuses Mo 3d orbits and (**b**) shows S 2p orbits

Additional study using XPS (Fig. 3) also proved the improvement of crystal quality and phase transition with the increase of temperature. The sharpening peaks from MG-150 to MG-240 indicate the crystal improves from a poly-state to a crystalized state. Also, a gradual shifting of the Mo 3d peaks can be observed from MG-180 to MG-240, and the binding energy of MG-180 appears ~0.63 eV lower than that of MG-240. This indicates that the possible crystal phase changes from 1 T to 2H from 180 °C to 240 °C [39, 41]. An insightful peak area calculation of Mo 3D peaks indicates the 2H to 1 T molar ratios vary from 4.84:1 (MG-150) to 3.01:1 (MG-180) and 13.7:1 (MG-210). For MG-240, no 1 T peaks can be deconvoluted. The peak positions of MG-150 are close to those of MG-210, which can be explained by the broad peaks with more lattice defects, and the loosely organized structure plays a more important role. Based on XRD and Raman data, crystallization quality and phase transition are two notable effects of temperature variation in the hydrothermal preparation of MoS_2_/graphene hybrids.

Previous studies reported that defects in crystals can increase the catalytic reaction rate, and a 1 T phase of MoS_2_ is always preferred. However, a significantly lower crystal quality leads to poorer charge transfer and lower catalytic performance [17, 42]. It is necessary to determine an optimum temperature to balance these factors. Additionally 1T phase of MoS_2_ is known to show limited stability in ambient environment [39, 41, 43], so its fraction in the hybrids are lower than the 2H phase for different preparation temperatures through XPS calculations. By optimizing the temperature, an optimized fraction of 1T phase can be determined in this facile hydrothermal method. Earlier studies also reported the mechanism of MoS_2_ formation, and the analyses are applicable here [12, 44]. Firstly, thiourea dissociates to free thiol groups and amino groups and reduces Mo(IV) and partially reduces GO. Secondly, free radicals adsorbed onto reduced GO surface start forming MoS_2_ crystals along the (002) face based on HRTEM results; defects are easier to form at a low temperature due to slower chemical kinetics, which exposes vacant Mo or S to the environment. Density functional theory (DFT) calculations show that reduction reactions tend to happen more along Mo-Mo grain boundaries than point defects in lattice [45], and the Mo-Mo grain boundaries are more abundant in low-temperature prepared defect-rich hybrids.

The importance of 1T phase of MoS_2_ for catalytic reactions has also been studied for pure 2D crystals. Earlier, DFT calculations suggest the 1T–MoS_2_ shows metallic properties and has a significantly higher catalytic reactivity compared with semiconducting 2H–MoS_2_ [39, 41, 46]. Studies also indicate the strong dependence of the crystal formation on temperature [47]. Pure 1T–MoS_2_ nanosheets are always prepared by chemical exfoliations by alkali metal [39], to obtain a higher ratio of 1T phase. Considering the costs and stabilities of 1T phase, hydrothermal methods are more suitable for catalytic reactions, which typically requires ~220 °C to have the best efficiency for pure MoS_2_ [34]. MoS_2_/graphene hybrids in this work, show lower temperature requirements at 180 °C, and this can be explained by the faster seeding process with graphene as the supporting media and crystal constant alignments during crystallization. A first-principle study of MoS_2_/graphene heterojunction shows that the work function of graphene (4.3 eV) matches well with the conduction band (4.2 eV) of monolayer MoS_2_, and the calculated charge carrier density in MG hybrids are over 3 orders of magnitude higher than the intrinsic value of graphene. Furthermore, the electron-hole pairs are well separated in the structure, which promotes a higher reactivity [21, 48, 49].

The electrocatalytic activity of MoS_2_/graphene hybrids were first investigated in DSSCs. DSSCs have a sandwich structure with a sensitizing material-coated semiconducting layer as the photoanode, a pair of redox as the electrolyte, and a reducing catalyst as the counter electrode [50]. DSSCs have separate photoanode and counter electrode, which creates an opportunity to maximize the counter electrode catalyst without breaking the cell chemistry. By applying the MoS_2_/graphene hybrid as the counter electrode in DSSCs, both the conductivity and the catalytic reactivity relevant to its electrochemical properties can be directly characterized.

**Fig. 4:**
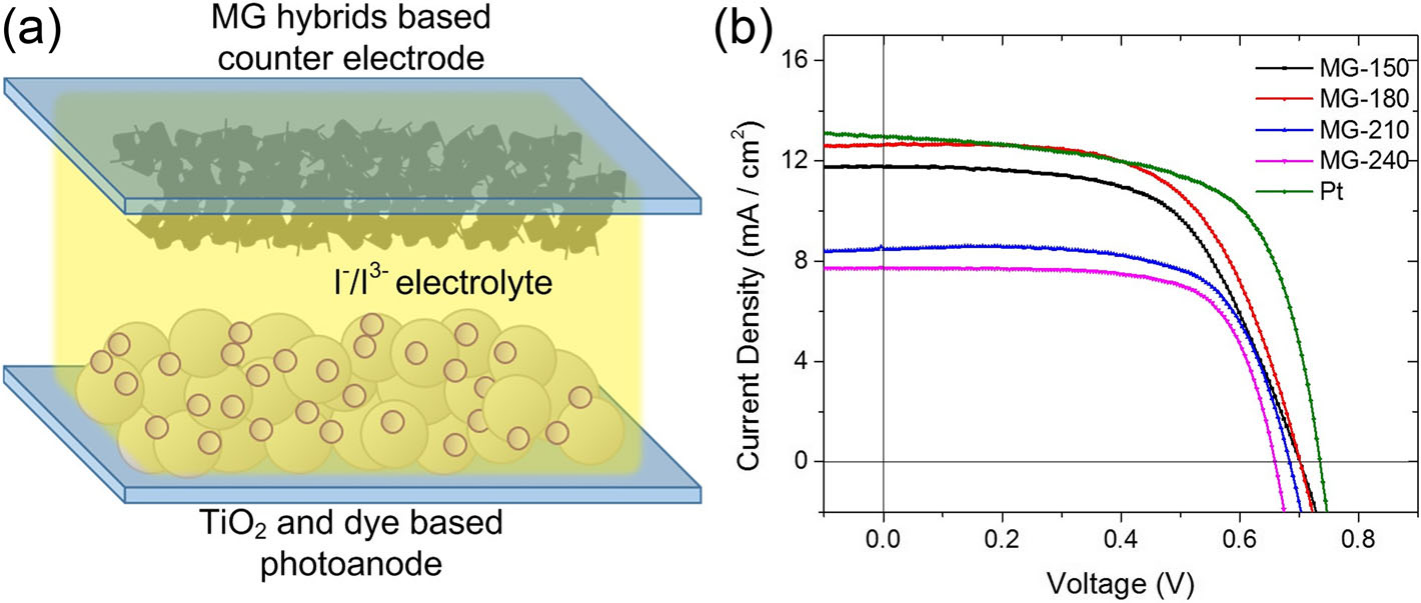
DSSC schematic and performance. **a** Schematic of the DSSC with as-prepared hybrids as the counter electrode catalyst. **b** J-V curves of DSSCs with MoS_2_/graphene hybrids as the counter electrode. The Pt-based counter electrode showed the best performance while the 180 °C hybrid was close to that with a lower FF. *V*_OC_ started dropping when the preparation temperature increased to 210 °C and 240 °C

In this work, we prepared N719-sensitized TiO_2_-based photoanode, I_3_^−^/I^−^ electrolyte, and MoS_2_/graphene hybrid counter electrodes for DSSC measurements, as shown in Fig. 4a. The solar cell performance is summarized in Table 1 and compared in Fig. 4b. Both MG-150 and MG-180 hybrids showed a significantly improved response compared with hybrids obtained at higher temperatures. All catalysts maintained the open-circuit voltage (*V*_OC_) at around 0.7 V, which is close to that of the Pt-based catalyst, while the short-circuit current (*i*_sc_) dropped to 8.47 mA/cm^2^ for MG-210 and 7.71 mA/cm^2^ for the MG-240 hybrids. The increased fill factor (FF) for high temperature hybrids results from the lower *i*_sc_ and *V*_OC_. It is clear that *i*_sc_ is the dominating factor for the efficiency that depends on fast charge transportation in the hybrids. Comparing the MG-150 and MG-180 catalysts, the MG-180 hybrid gave a higher *i*_sc_, which suggests either a better conductivity or a higher reactivity, and agrees well with the weakened charge transportation prediction by excessive defects in the MG-150 hybrid. The low performance of the MG-240 hybrid is predictable because of the over-stacking of MoS_2_ nanosheets, shown in SEM images of Fig. 1, which limits the electron transfer between the graphene and MoS_2_ crystals. A further investigation on the resistance through EIS analysis (Additional file 1: Figure S3) suggests the lowest charge transfer resistance of MG-180, which agrees well with the efficiency performance.

**Table 1:** DSSC performance data comparison

Counter electrode	*V*_OC_ (V)	*i*_sc_ (mA/cm^2^)	FF (%)	Efficiency (%)
MG-150	0.700	11.7	59.2	4.87
MG-180	0.701	12.6	60.4	5.34
MG-210	0.683	8.47	67.4	3.90
MG-240	0.659	7.71	70.2	3.57
Pt	0.735	13.0	64.0	6.10

To further understand the improved performance of the MG-180 hybrid in DSSCs, the conductivity and reactivity must be investigated separately. To study the electrochemical properties, the MG-150, MG-180, and MG-210 hybrids were chosen to measure the HER performance in a three-electrode setup. All HER tests were operated in the 0.5 M H_2_SO_4_ aqueous solution using an Ag/AgCl electrode as the reference and Pt wire as the counter electrode. The electrochemical performance of samples was tested by fabricating glassy carbon electrodes with a controlled diameter of 3 mm, and the tested potentials were converted to a RHE.

**Fig. 5:**
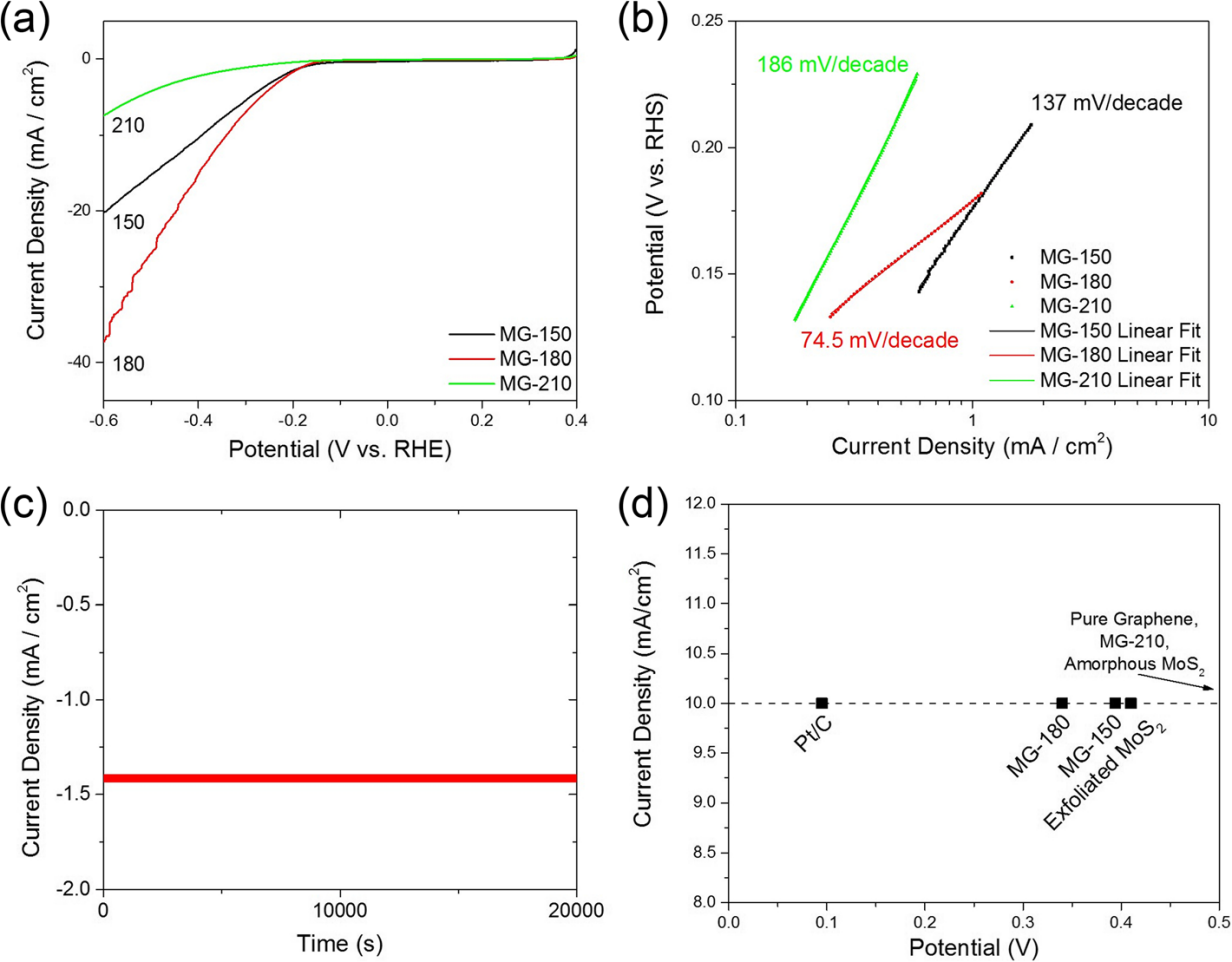
HER performance comparison. **a** Polarization curves after IR correction. **b** Corresponding Tafel plots of MoS_2_/graphene hybrids prepared at 150, 180, and 210 °C, **c** I-t scan of MG-180 for 20,000 s. **d** Comparison of overpotentials at 10 mA/cm^2^ for MG-150, MG-180, and MG-210 with Pt/C, exfoliated MoS_2_, and amorphous MoS_2_

The MG-150 and MG-180 hybrids gave very close onset potentials of about −176 and −179 mV, respectively, and the MG-210 showed an onset potential about −287 mV, estimated from the low-current density region in the LSV (Fig. 5a). The shaking tail of the MG-180 hybrid at a lower potential was caused by the generation and accumulation of hydrogen bubbles, which suggests the high performance of the MoS_2_/graphene hybrid. The Tafel plots (Fig. 5b) of three catalysts show a 74.5 mV/decade slope for the MG-180 hybrid, which is much lower than those of MG-150 and MG-210, indicating a faster increase of the HER rate with increasing overpotentials. The better performance of the MG-180 hybrid over the MG-150 hybrid explains the importance of better crystallization for charge transfer. This can be observed by EIS analysis (Additional file 1: Figure S5). The MG-180 hybrid exhibited a smaller semicircle, indicating more efficient charge transfer between graphene and MoS_2_. Meanwhile, the impedance of the MG-180 hybrid quickly increased, presenting the possibility of higher porosity of the same mass of materials. Brunauer-Emmett-Teller (BET) tests indicated that MG-180 has a specific surface area of 73.5 m^2^/g, compared with those of MG-150 (49.5 m^2^/g) and MG-210 (73.4 m^2^/g). The result agrees well with the highly branched structures shown in the SEM images. The Tafel slope of 137 mV/decade for the MG-150 hybrid also explains its slightly lower efficiency in DSSCs. CV results (Additional file 1: Figure S4) showed that the MG-180 hybrid has a larger difference of reduction/oxidation potential and a higher peak current, suggesting more active sites in MG-180 hybrids and higher reactivity in electrochemical reactions.

Besides the HER reactivity of the MG-180 hybrid, a stable performance was also demonstrated by a constant potential of −0.5 V for 20,000 s (Fig. 5c). A comparison of as-prepared hybrids with exfoliated MoS_2_ and amorphous MoS_2_ performance at the same current density highlights the outperformance of the MG-180 with a lower overpotential (Fig. 5d) [3, 51]. Therefore, 180 °C offers a preferred balance of the active defect sites, 1T phase of MoS_2_ and branched structures for catalytic activities.

## Conclusions

In summary, the crystallization condition of MoS_2_/graphene hybrids was studied by structure characterizations and performance measurements of DSSC and HER. Benefiting from the excellent reactivity of MoS_2_ and high conductivity of graphene, the hybrids show stable and improved performance compared with their constituents. The MoS_2_ in the hybrid shows a crystal phase change from 1T in the low-temperature region (below 180 °C) to 2H in the high-temperature region (above 210 °C), along with crystal quality improvement and reduced defect sites. The existence of the 1T phase improves the reduction reactivity and charge transfer ability of the hybrid. The controlled defect sites also improve the catalytic reaction rate. The morphology of MoS_2_ on graphene is essential for maintaining high catalytic performance and perpendicularly oriented structures in flower-like shape is preferred. This work provides a fundamental guideline and understanding for the rational design and construction of 2D hybrid materials for electrocatalytic applications.

## Additional file

Additional file 1: Supporting Information for Temperature-dependent Crystallization of MoS_2_ Nanoflakes on Graphene Nanosheets for Electrocatalysis. (DOCX 1160 kb)

## Abbreviations

2D: Two-dimensional
3D: Three-dimensional
BET: Brunauer-Emmett-Teller
CV: Cyclic voltammetry
DFT: Density functional theory
DSSC: Dye-sensitized solar cell
EDS: Energy-dispersive X-ray spectroscopy
EIS: Electrochemical impedance spectroscopy
FE-SEM: Field-emission scanning electron microscope
FF: Fill factor
GCE: Glassy carbon electrode
HER: Hydrogen evolution reaction
HRTEM: High-resolution transmission electron microscope
i_sc_: Short-circuit current
LSV: Linear sweep voltammetry
MEGO: Microwave-exfoliated graphene oxide nanosheets
RHE: Reversible hydrogen electrode
SAED: Selected area electron diffraction
TEM: Transmission electron microscope
TMD: Transition metal dichalcogenide
V_OC_: Open-circuit voltage
XPS: X-ray photoelectron spectroscopy
XRD: X-ray diffraction

## References

[1] Xu M, Liang T, Shi M, Chen H (2013). Graphene-like two-dimensional materials. Chem Rev.

[2] Mao S, Wen Z, Ci S, Guo X, Ostrikov KK, Chen J (2015). Perpendicularly oriented MoSe2 /graphene nanosheets as advanced electrocatalysts for hydrogen evolution. Small.

[3] Hou Y, Zhang B, Wen ZH, Cui SM, Guo XR, He Z (2014). A 3D hybrid of layered MoS2/nitrogen-doped graphene nanosheet aerogels: an effective catalyst for hydrogen evolution in microbial electrolysis cells. J Mater Chem A.

[4] Hou Y, Wen Z, Cui S, Guo X, Chen J (2013). Constructing 2D porous graphitic C3 N4 nanosheets/nitrogen-doped graphene/layered MoS2 ternary nanojunction with enhanced photoelectrochemical activity. Adv Mater.

[5] David L, Bhandavat R, Singh G (2014). MoS2/graphene composite paper for sodium-ion battery electrodes. ACS Nano.

[6] Chang K, Chen W (2011). L-cysteine-assisted synthesis of layered MoS(2)/graphene composites with excellent electrochemical performances for lithium ion batteries. ACS Nano.

[7] Liu GB, Shan WY, Yao YG, Yao W, Xiao D (2013). Three-band tight-binding model for monolayers of group-VIB transition metal dichalcogenides. Phys Rev B.

[8] Prins F, Goodman AJ, Tisdale WA (2014). Reduced dielectric screening and enhanced energy transfer in single- and few-layer MoS2. Nano Lett.

[9] Debbichi L, Eriksson O, Lebegue S (2014). Electronic structure of two-dimensional transition metal dichalcogenide bilayers from ab initio theory. Phys Rev B.

[10] Zhou W, Zou X, Najmaei S, Liu Z, Shi Y, Kong J (2013). Intrinsic structural defects in monolayer molybdenum disulfide. Nano Lett.

[11] Novoselov KS, Jiang D, Schedin F, Booth TJ, Khotkevich VV, Morozov SV (2005). Two-dimensional atomic crystals. Proc Natl Acad Sci U S A.

[12] Li Y, Wang H, Xie L, Liang Y, Hong G, Dai H (2011). MoS2 nanoparticles grown on graphene: an advanced catalyst for the hydrogen evolution reaction. J Am Chem Soc.

[13] Wang H, Yuan H, Sae Hong S, Li Y, Cui Y (2015). Physical and chemical tuning of two-dimensional transition metal dichalcogenides. Chem Soc Rev.

[14] Patil SA, Kalode PY, Mane RS, Shinde DV, Doyoung A, Keumnam C (2014). Highly efficient and stable DSSCs of wet-chemically synthesized MoS2 counter electrode. Dalton Trans.

[15] Finn ST, Macdonald JE (2014). Petaled molybdenum disulfide surfaces: facile synthesis of a superior cathode for QDSSCs. Adv Energy Mater.

[16] Xie J, Zhang J, Li S, Grote F, Zhang X, Zhang H (2013). Controllable disorder engineering in oxygen-incorporated MoS2 ultrathin nanosheets for efficient hydrogen evolution. J Am Chem Soc.

[17] Lukowski MA, Daniel AS, Meng F, Forticaux A, Li L, Jin S (2013). Enhanced hydrogen evolution catalysis from chemically exfoliated metallic MoS2 nanosheets. J Am Chem Soc.

[18] Stankovich S, Dikin DA, Piner RD, Kohlhaas KA, Kleinhammes A, Jia Y (2007). Synthesis of graphene-based nanosheets via chemical reduction of exfoliated graphite oxide. Carbon.

[19] Dikin DA, Stankovich S, Zimney EJ, Piner RD, Dommett GH, Evmenenko G (2007). Preparation and characterization of graphene oxide paper. Nature.

[20] Geim AK, Novoselov KS (2007). The rise of graphene. Nat Mater.

[21] Hu W, Yang JL (2016). First-principles study of two-dimensional van der Waals heterojunctions. Comput Mater Sci.

[22] Deng D, Novoselov KS, Fu Q, Zheng N, Tian Z, Bao X (2016). Catalysis with two-dimensional materials and their heterostructures. Nat Nanotechnol.

[23] Jeffery AA, Rao SR, Rajamathi M (2017). Preparation of MoS2–reduced graphene oxide (rGO) hybrid paper for catalytic applications by simple exfoliation–costacking. Carbon.

[24] Pierucci D, Henck H, Avila J, Balan A, Naylor CH, Patriarche G (2016). Band alignment and Minigaps in monolayer MoS2-Graphene van der Waals Heterostructures. Nano Lett.

[25] Toth PS, Velicky M, Bissett MA, Slater TJ, Savjani N, Rabiu AK (2016). Asymmetric MoS2 /Graphene/metal sandwiches: preparation, characterization, and application. Adv Mater.

[26] Zhang JM, Zhao L, Liu AP, Li XY, Wu HP, Lu CD (2015). Three-dimensional MoS2/rGO hydrogel with extremely high double-layer capacitance as active catalyst for hydrogen evolution reaction. Electrochim Acta.

[27] Lin JY, Su AL, Chang CY, Hung KC, Lin TW (2015). Molybdenum disulfide/reduced Graphene oxide-carbon Nanotube hybrids as efficient catalytic materials in dye-sensitized solar cells. Chem Aust.

[28] Shi J, Zhou X, Han G-F, Liu M, Ma D, Sun J (2016). Narrow-gap quantum wires arising from the edges of monolayer MoS2Synthesized on Graphene. Adv Mater Interfaces.

[29] Firmiano EG, Cordeiro MA, Rabelo AC, Dalmaschio CJ, Pinheiro AN, Pereira EC (2012). Graphene oxide as a highly selective substrate to synthesize a layered MoS2 hybrid electrocatalyst. Chem Commun.

[30] Zheng XL, Xu JB, Yan KY, Wang H, Wang ZL, Yang SH (2014). Space-confined growth of MoS2 Nanosheets within graphite: the layered hybrid of MoS2 and Graphene as an active catalyst for hydrogen evolution reaction. Chem Mater.

[31] Dong H, Liu C, Ye H, Hu L, Fugetsu B, Dai W (2015). Three-dimensional nitrogen-doped Graphene supported molybdenum disulfide Nanoparticles as an advanced catalyst for hydrogen evolution reaction. Sci Rep.

[32] Xue N, Diao P. Composite of Few-Layered MoS2 Grown on Carbon Black: Tuning the Ratio of Terminal to Total Sulfur in MoS2 for Hydrogen Evolution Reaction. J Phys Chem. 2017;121(27):14413-25.

[33] Zhang HP, Lin HF, Zheng Y, Hu YF, MacLennan A (2015). Understanding of the effect of synthesis temperature on the crystallization and activity of nano-MoS2 catalyst. Appl Catal B-Environ.

[34] Wang DZ, Pan Z, Wu ZZ, Wang ZP, Liu ZH (2014). Hydrothermal synthesis of MoS2 nanoflowers as highly efficient hydrogen evolution reaction catalysts. J Power Sources.

[35] Zhu YW, Murali S, Stoller MD, Velamakanni A, Piner RD, Ruoff RS (2010). Microwave assisted exfoliation and reduction of graphite oxide for ultracapacitors. Carbon.

[36] Ito S, Murakami TN, Comte P, Liska P, Grätzel C, Nazeeruddin MK (2008). Fabrication of thin film dye sensitized solar cells with solar to electric power conversion efficiency over 10%. Thin Solid Films.

[37] Hou Y, Cui S, Wen Z, Guo X, Feng X, Chen J (2015). Strongly coupled 3D hybrids of N-doped porous carbon Nanosheet/CoNi alloy-encapsulated carbon Nanotubes for enhanced Electrocatalysis. Small.

[38] Hashimoto A, Suenaga K, Gloter A, Urita K, Iijima S (2004). Direct evidence for atomic defects in graphene layers. Nature.

[39] Acerce M, Voiry D, Chhowalla M (2015). Metallic 1T phase MoS2 nanosheets as supercapacitor electrode materials. Nat Nanotechnol.

[40] Chen JM, Wang CS (1974). Second order Raman spectrum of MoS2. Solid State Commun.

[41] Ambrosi A, Sofer Z, Pumera M (2015). 2H --> 1T phase transition and hydrogen evolution activity of MoS2, MoSe2, WS2 and WSe2 strongly depends on the MX2 composition. Chem Commun.

[42] Yan Y, Ge X, Liu Z, Wang JY, Lee JM, Wang X (2013). Facile synthesis of low crystalline MoS2 nanosheet-coated CNTs for enhanced hydrogen evolution reaction. Nano.

[43] Wang L, Liu X, Luo J, Duan X, Crittenden J, Liu C (2017). Active site self-optimization by irreversible phase transition of 1T-MoS2 in Photocatalytic hydrogen evolution. Angew Chem Int Ed.

[44] Hansen LP, Johnson E, Brorson M, Helveg S (2014). Growth mechanism for single- and multi-layer MoS2Nanocrystals. J Phys Chem C.

[45] Ouyang Y, Ling C, Chen Q, Wang Z, Shi L, Wang J (2016). Activating inert basal planes of MoS2for hydrogen evolution reaction through the formation of different intrinsic defects. Chem Mater.

[46] Akashi R, Ochi M, Bordács S, Suzuki R, Tokura Y, Iwasa Y (2015). Two-Dimensional Valley electrons and Excitons in Noncentrosymmetric 3R-MoS2. Phys Rev Appl.

[47] Duerloo KA, Reed EJ (2016). Structural phase transitions by Design in Monolayer Alloys. ACS Nano.

[48] Britnell L, Ribeiro RM, Eckmann A, Jalil R, Belle BD, Mishchenko A (2013). Strong light-matter interactions in heterostructures of atomically thin films. Science.

[49] Myoung N, Seo K, Lee SJ, Ihm G (2013). Large current modulation and spin-dependent tunneling of vertical graphene/MoS2 heterostructures. ACS Nano.

[50] Guo X, Lu G, Chen J (2015). Graphene-based materials for Photoanodes in dye-sensitized solar cells. Front Energy Res.

[51] Eng AY, Ambrosi A, Sofer Z, Simek P, Pumera M (2014). Electrochemistry of transition metal dichalcogenides: strong dependence on the metal-to-chalcogen composition and exfoliation method. ACS Nano.

