# Supplementary material for: Temperature-dependent Crystallization of MoS2 Nanoflakes on Graphene Nanosheets for Electrocatalysis
Source: Nanoscale Res Lett. 2017 Aug 4;12:479. doi: 10.1186/s11671-017-2248-9 (PMC5544664; doi:10.1186/s11671-017-2248-9)
Supplement: Additional file 1: — Supporting Information for Temperature-dependent Crystallization of MoS2 Nanoflakes on Graphene Nanosheets for Electrocatalysis. (DOCX 1160 kb) [file 11671_2017_2248_MOESM1_ESM.docx]

**Supporting Information**

**Temperature-dependent Crystallization of MoS_2_ Nanoflakes on Graphene Nanosheets for Electrocatalysis**

Xiaoru Guo^1^, Yang Hou^1^, Ren Ren^1^, Junhong Chen^1*^

^1^Department of Mechanical Engineering, University of Wisconsin-Milwaukee, Milwaukee, WI, USA.

***Corresponding:** Department of Mechanical Engineering, University of Wisconsin-Milwaukee, 3200 North Cramer Street, Milwaukee, WI, 53211, USA. [jhchen@uwm.edu](mailto:jhchen@uwm.edu)

### Experimental Details

1. Materials

Carbon powders for GO synthesis were purchased from Bay Carbon, Inc. (Part No.: SP-1). Commercial TiO_2_ paste SA (Part No.: Ti-Nanoxide T/SP), iodine electrolyte (Part No.: Iodolyte HI-30) and thermoplastic sealing film (Part No.: Meltonix 1170-25) were purchased from Solaronix. All other materials were purchased from Sigma-Aldrich.

1. Preparation of MoS_2_ on graphene nanosheets

Graphene oxide nanosheets were prepared according to published procedures. In short, graphene oxide (GO) was first synthesized from a modified Hummer’s method.(1) After washing and drying the as-prepared GO at 65 ^o^C for 48 h, the solid flakes were then exfoliated and reduced by microwave at 900 W for 90 s under an argon environment.(2)

To prepare the MoS_2_, 10 wt.% graphene (2.8 mg) was dispersed in 20 mL DI water by ultra-sonication at 250 W for 30 min. Then, sodium molybdate dihydrate (Na_2_MoO_4_·2H_2_O, 42 mg) was added in and stirred for 30 min, to be well-absorbed onto the MEGO surface. Finally, thiourea (SC(NH_2_)_2_, 84 mg) was added and stirred for 30 min, and the solution was transferred to a 50 mL autoclave for hydrothermal reaction at temperatures that ranged from 150 ^o^C to 240 ^o^C. The reaction can be written as

4 MoO_4_^2-^ + 9 SC(NH_2_)_2_ + 6 H^+^ → 4 MoS_2_ + 9 OC(NH_2_)_2_ + SO_4_^2-^ + 3 H_2_O

The molar ratio of Mo source to S source required for the reaction is close to 1:2.25. However, in our work the molar ratio of Mo source to S source is about 1:10; so thiourea is excessive in this work.

After the synthesis, the obtained suspensions were transferred to 50 mL centrifuge tubes. The solids were separated after standing steadily for one day and then centrifuged at 11,000 rpm for 40 min. The MoS_2_/graphene hybrids were obtained after washing with DI water three times and then dried under vacuum at 70 ^o^C overnight.

1. Material characterization

The structure of prepared materials was studied with a Hitachi (S-4800) scanning electron microscope (FE-SEM). The EDS mapping data were obtained using a Bruker detector on a Hitachi S-4800. A Hitachi (H 9000 NAR) transmission electron microscope (TEM/HR-TEM) was used to study the hybrid junction of the MoS_2_/graphene hybrid prepared at 180 ^o^C. XRD was done using a Bruker D8 Discover X-ray diffractometer. Raman spectroscopy was taken with a Renishaw Raman spectrometer (Inc 1000B) with an HeNe laser (633 nm).

1. DSSC fabrication

First, FTO glasses were sequentially cleaned with acetone, isopropyl alcohol, and DI water. Then, the glass blades were fixed onto the bench with Scotch^®^ tape and a commercial TiO_2_ paste was doctor-bladed onto the surface. After gradually heating to 500 ^o^C over 30 min, the substrates were treated in a 40 mM TiCl_4_ aqueous solution for 30 min at 70 ^o^C, and then dried at 500 ^o^C for 30 min. After cooling down to room temperature, the substrates were transferred to 0.5 mM N719 ethanol solution and soaked for 24 h. Detailed steps can be found in earlier publications.(3)

The counter electrodes were also fabricated by doctor-blading. First, a 20 mg sample and 5 µL Triton x100 was dispersed in 500 µL DI water. Then the obtained slurry was bladed onto a clean FTO surface and then annealed at 500 ^o^C for 30 min in an argon environment. Pt-based counter electrodes were fabricated by blading 0.01 M H_2_PtCl_6_ ethanol solution with the same steps.

To assemble the cell, the prepared counter electrodes and photoanodes were sealed with a commercial thermoplastic sealing film, and then a commercial electrolyte was injected between the cells.

1. DSSC test

The J-V characterization was conducted under a simulated one sun illumination (AM 1.5G, 100 mW/cm^2^, Newport, 94021A) with a Keithley 2420 source meter. The system was calibrated with an Si-reference cell (Oriel, P/N 91150V).

1. Electrochemical measurements

A saturated Ag/AgCl reference electrode was used in all measurements and converted to the reversible hydrogen electrode (RHE) scale via the Nernst equation. All measurements were carried out in 0.5 M H_2_SO_4_ aqueous solution using a CHI 760D electrochemical workstation. Tests were performed in a standard three-electrode glass cell, with the Pt wire as the counter electrode and glassy carbon electrode (GCE). To fabricate GCEs, 5 mg of material was mixed with 50$\mu L$ Nafion ethanol solution (5%) and 450$\mu L$ DI water. The mixture was well dispersed and a 5$\mu L$ suspension was dropped onto a glassy carbon electrode with a diameter of 3 mm and then fully dried.

The linear sweep voltammetry (LSV) was tested from 0.2 to -0.8 V (vs. Ag/AgCl) at 5 mV/s; later the Tafel plot was calculated from LSV. Cyclic Voltammetry was scanned between -1 V and 1 V (vs. Ag/AgCl) at 0.05$V/s$. The electrochemical impedance spectroscopy (EIS) was measured at a frequency ranging from 0.1 to 10,000 Hz at a constant potential 0.5 mV (vs. Ag/AgCl). The stability was evaluated for 20,000 s at a constant potential -0.5 V (vs. Ag/AgCl).

1. Sheet resistance measurement

A 1 inch by 1 inch square glass slide was used as the substrate. 50 µL solution of the electrochemical mixture for each sample was drop-casted onto the glass slide, and gently spread to form a uniform surface by the surface tension of the liquid. After drying in the ambient lab environment, a uniform film was obtained for sheet resistance measurements. Due to the low density and high static electricity of the as-prepared hybrids, a stamped film cannot be obtained. The obtained film was tested by a 4-probe sheet resistance measurement system, using a Keithley 2420 source meter.

### Additional Results


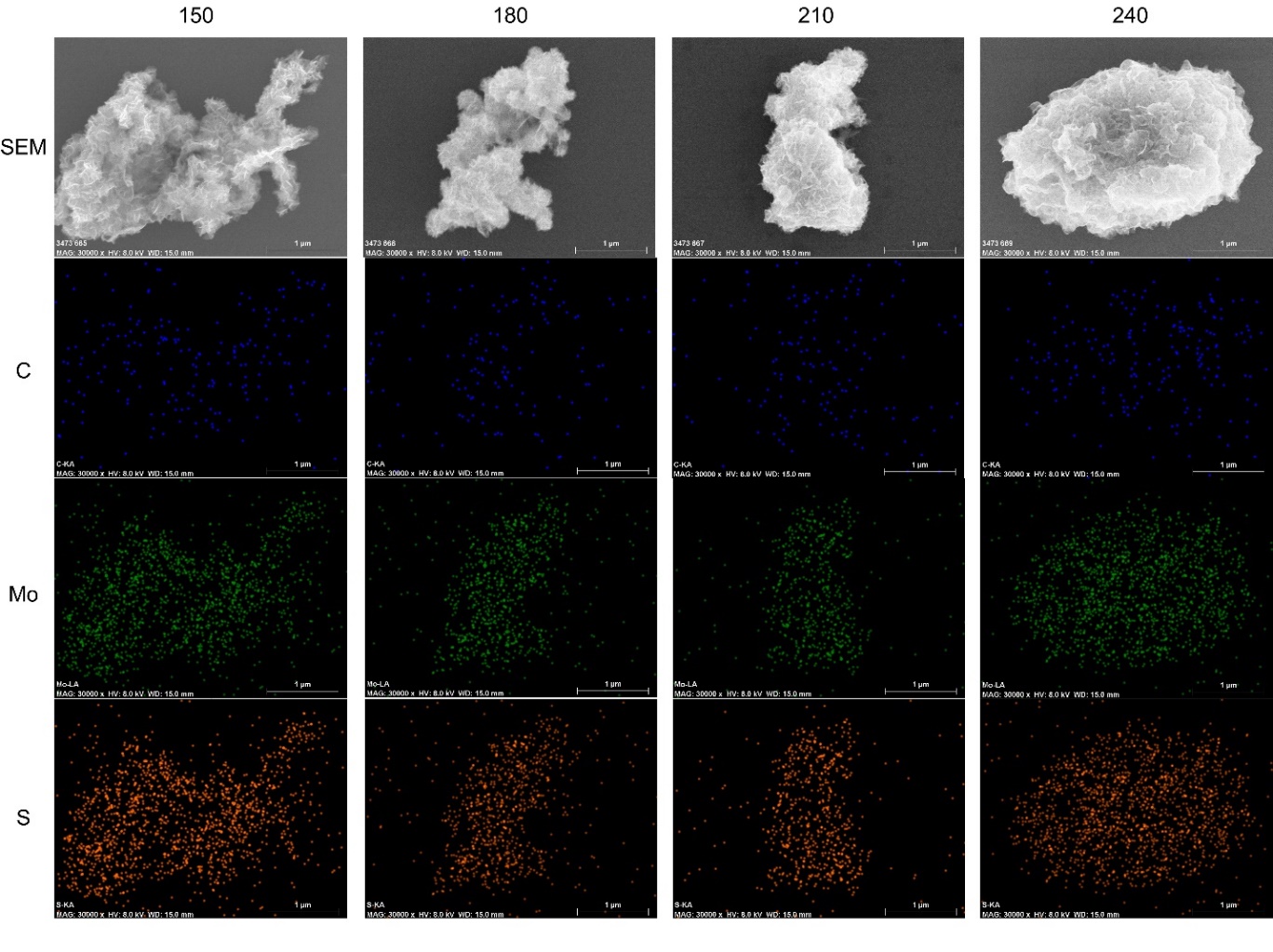


Figure S1. EDS results with SEM images for four samples at different temperatures. The distributions of C, Mo, and S elements are uniform, proving the complete coverage of MoS_2_ on a graphene surface.


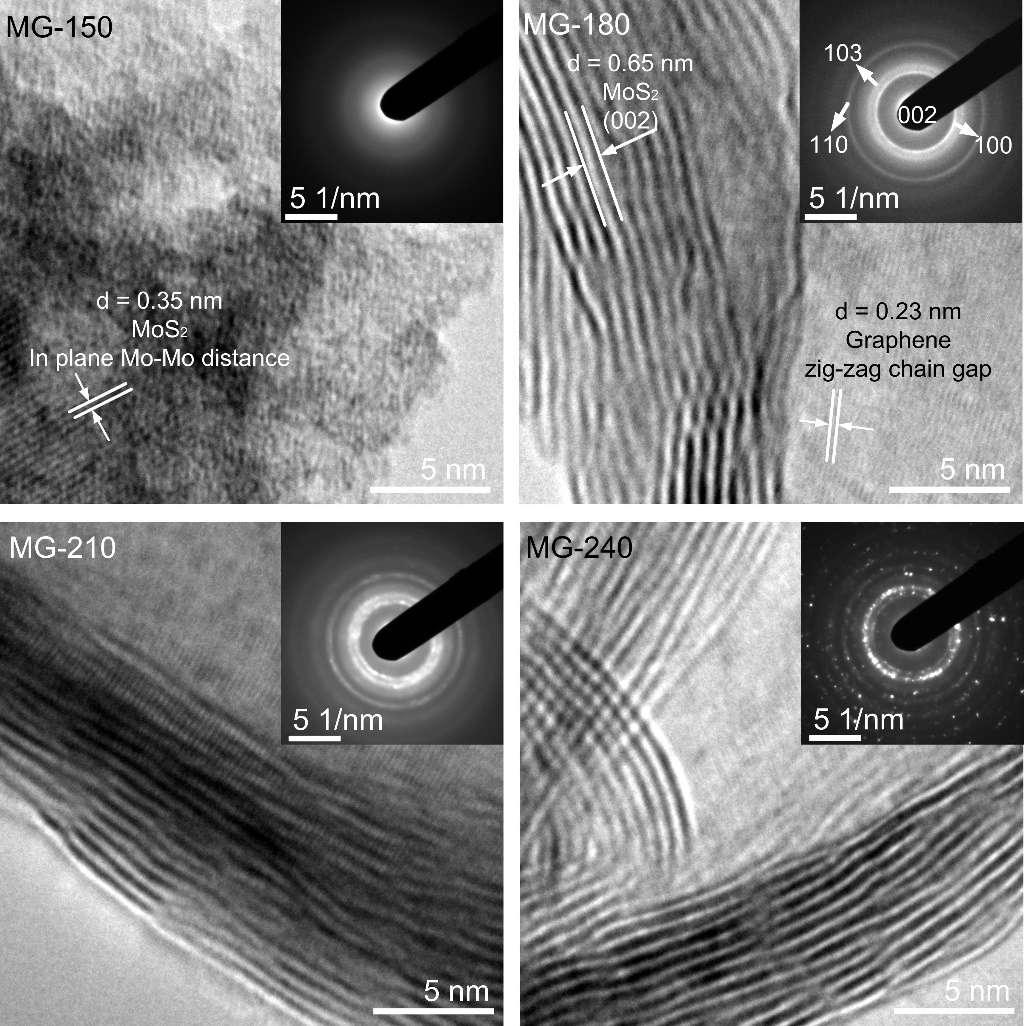


Figure S2. HRTEM and SAED results of all four hybrids. In the MG-150, only the in-plane Mo-Mo distance can be observed, which indicates a flat heterojunction. In MG-180, MG-210 and MG-240 hybrids, the thick stripes suggest the formation of layer-by-layer perpendicular structures of MoS_2_ nanosheets. The crystallized SAED dots can be observed with an increasing temperature, which indicates the improvement of crystallization at a higher temperature.


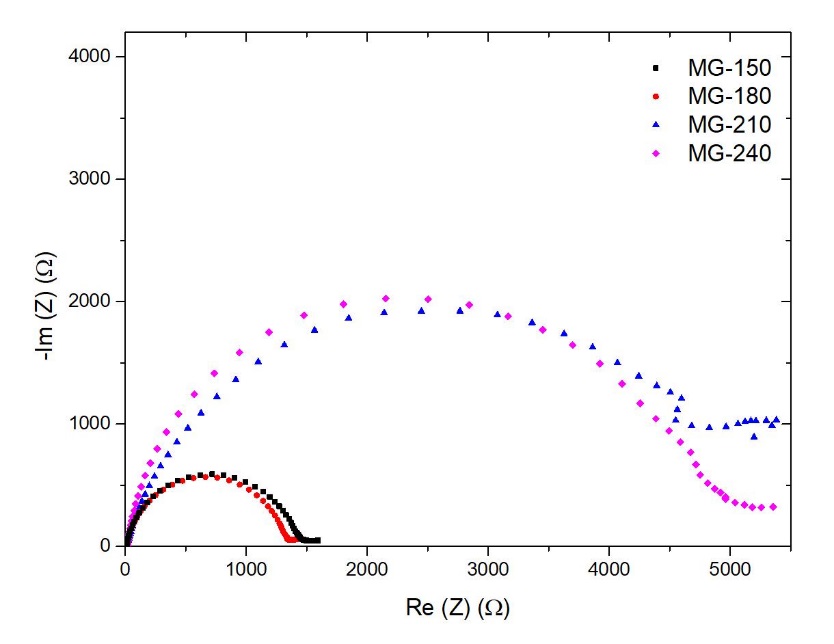


Figure S3. Nyquist plot of the EIS results of the as-prepared DSSCs. A typical Nyquist plot of DSSCs should contain three semi-circles, but in this work, the resistance in the hybrid material is significantly higher than other parts, and the resistance is dominated by the counter electrode resistance. Nyquist plots suggest the lowest resistance of MG-180, which agrees well with the efficiency data.


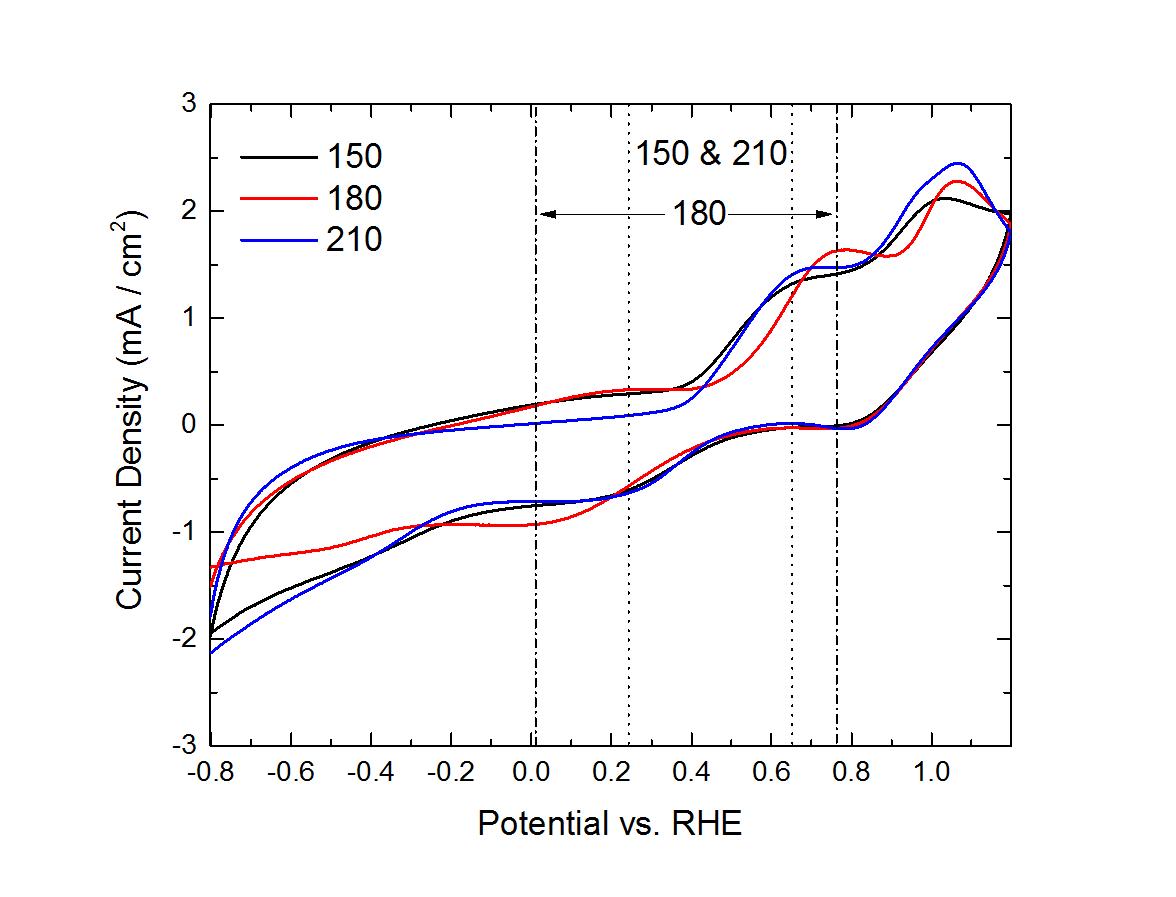


Figure S4. Cyclic voltammetry scans of MoS_2_/graphene hybrids prepared at 150 ^o^C, 180 ^o^C, and 210 ^o^C. The MG-180 hybrid gives a larger potential between the reduction and oxidation peaks than at 150 ^o^C and 210 ^o^C, indicating a more reliable reduction. At the same time, the higher current density at each peak indicates high reactivity, which likely results from more active sites in the 180 ^o^C hybrids.


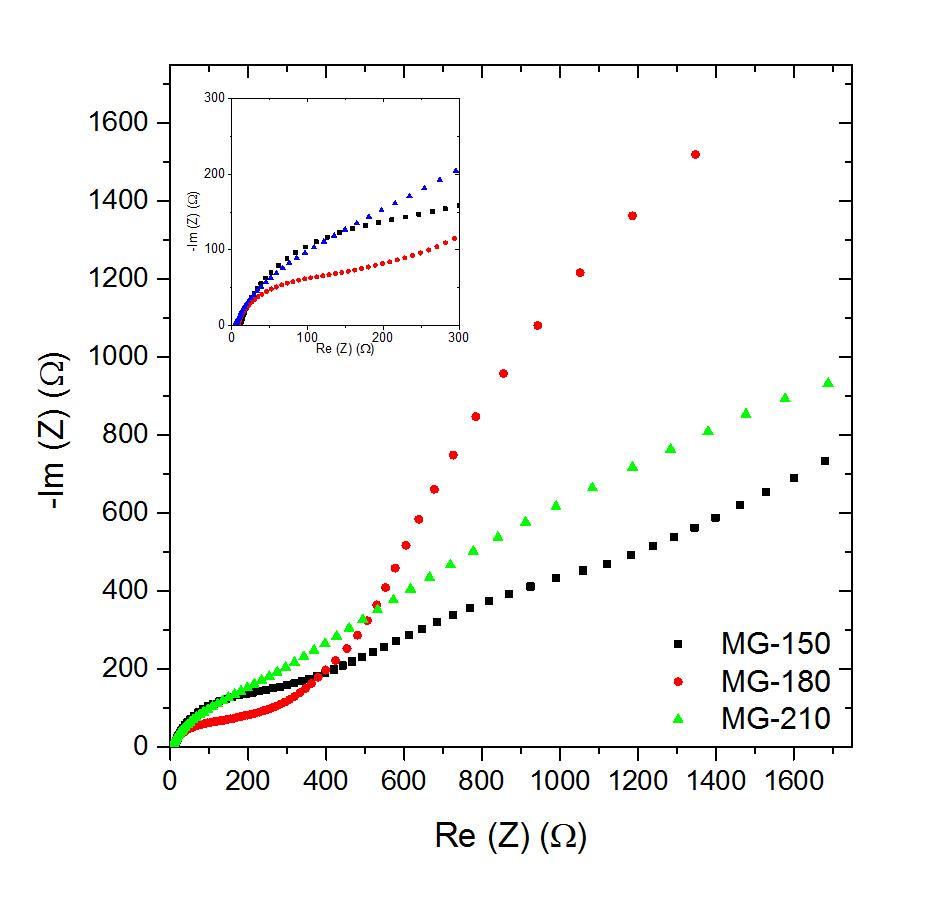


Figure S5. EIS Nyquist plots of MoS_2_/graphene hybrids prepared at 150 ^o^C, 180 ^o^C, and 210 ^o^C, with zoom-in view shown in the inset. The MG-180 hybrid exhibited a smaller semicircle and higher impedance, indicating faster charge transfer in the structure and higher porosity of the same mass of materials.


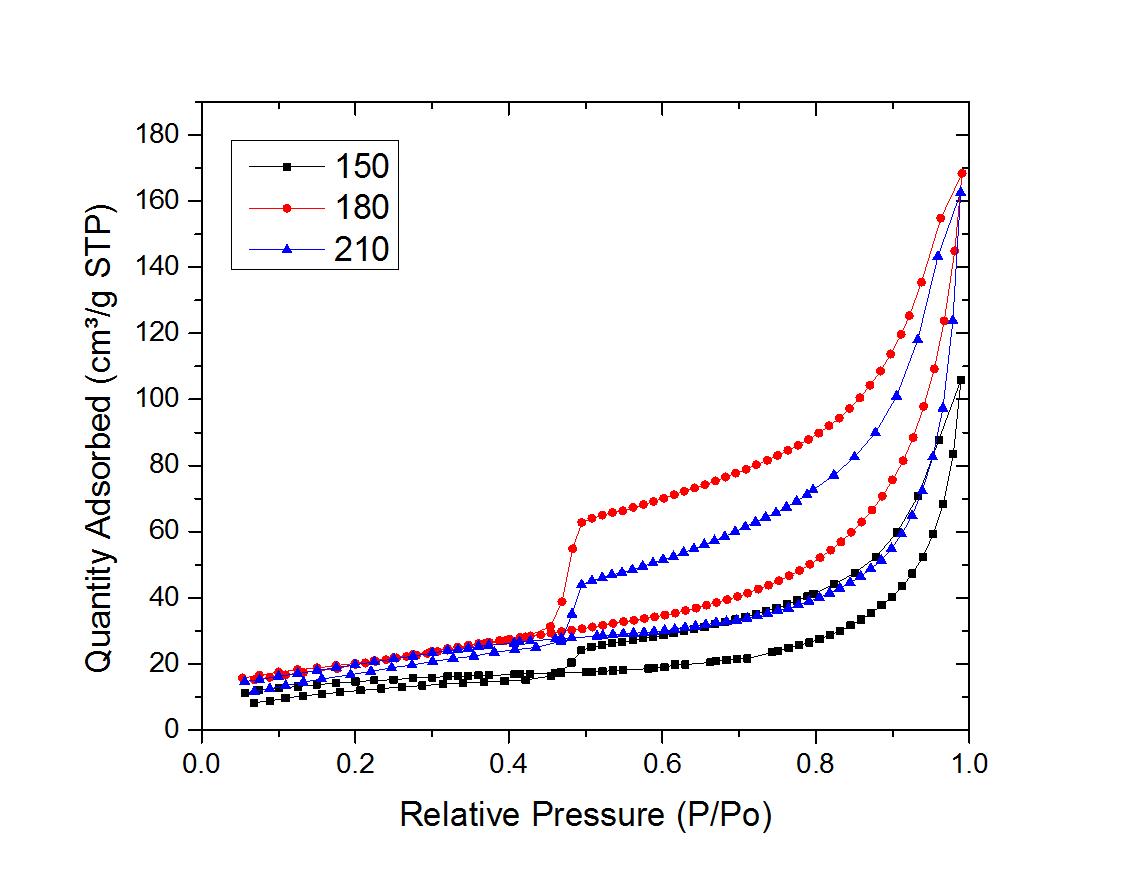


Figure S6. BET isotherm linear plot of MoS_2_/graphene hybrids prepared at 150 ^o^C, 180 ^o^C, and 210 ^o^C. Similar adsorbed quantity at P/P_0_= 1 of MG-180 and MG-210 can be understood by the similar surface profile, while the MG-210 hybrid has more stacking which leads to small pores and shows less mesopore effect on the trace of adsorption and desorption. The low surface area of the MG-150 hybrid proves that MG-150 hybrid has fewer layers and less active sites than that of high temperature ones.

Table S1 Sheet resistance of all four hybrids

| Hybrids | MG-150 | MG-180 | MG-210 | MG-240 |
| --- | --- | --- | --- | --- |
| Sheet resistance  (MΩ) | 101.2 | 29.8 | 123.8 | 185.8 |

### References

1. Marcano DC, Kosynkin DV, Berlin JM, Sinitskii A, Sun Z, Slesarev A, et al. Improved Synthesis of Graphene Oxide. ACS Nano. 2010;4(8):4806-14.

2. Zhu YW, Murali S, Stoller MD, Velamakanni A, Piner RD, Ruoff RS. Microwave assisted exfoliation and reduction of graphite oxide for ultracapacitors. Carbon. 2010;48(7):2118-22.

3. Ito S, Murakami TN, Comte P, Liska P, Grätzel C, Nazeeruddin MK, et al. Fabrication of thin film dye sensitized solar cells with solar to electric power conversion efficiency over 10%. Thin Solid Films. 2008;516(14):4613-9.
